# Case Report: A case study and literature review on teeth discoloration caused by linezolid with the shortest incubation period in a pediatric patient

**DOI:** 10.3389/fped.2024.1440322

**Published:** 2024-10-15

**Authors:** Sun Le-le, Zhao Qun, Qi Lei, Meng Xiangwei, Si Jigang

**Affiliations:** ^1^ Department of Pharmacy, Binzhou Medical University Affiliated Zibo Central Hospital, Zibo, Shandong, China; ^2^ Department of Medicinal Chemistry, School of Pharmacy, Qingdao University, Qingdao, China

**Keywords:** linezolid, adverse reactions, teeth discoloration, children, literature review

## Abstract

**Background:**

When it comes to the adverse reactions of linezolid, people always call to mind primarily nausea, vomiting, bone marrow suppression, and so on. Few people are aware of the rare adverse reaction of teeth discoloration.

**Case presentation:**

We describe the case of a child affected by bacterial meningitis. After admission, a combination of ceftriaxone and linezolid was administered for anti-infection, and dexamethasone was used to inhibit inflammatory reactions. On the 5th day of treatment with linezolid, the child's teeth appeared brownish color and could not be removed with normal oral hygiene. Upon reviewing the drug instructions and literature, it was found that the discoloration of teeth is a rare adverse reaction of linezolid, which is pseudo discoloration. After stopping the medication for 28 days or up to 5 months, the normal color can be restored. There is no significant impact on the life of the patient, therefore, continue to use linezolid to complete the anti-infection course.

**Results:**

After 14 days of anti-infection treatment, the inflammatory indicators of the child decreased to normal, and the condition was close to recovery before discharge. After stopping the medication for 28 days, the color of the teeth returned to normal.

**Conclusions:**

This rare adverse reaction sheds light on a previously unreported side effect of this widely used antibiotic. In our case, the discoloration of the teeth occurred earlier, updating the latent period of the adverse reaction.

## 1 Introduction

Linezolid is a synthetic oxazolidinone antimicrobial commonly employed for the treatment of Gram-positive bacterial infections. Its chemical structure distinguishes it from other antimicrobial agents, and its mechanism of action is unique due to its specific target in inhibiting bacterial protein synthesis. Linezolid primarily binds to the 23 s site of ribosomal RNA on the 50 s subunit of bacteria near the interface with the 30 s subunit, thereby preventing the formation of the 70 s initiation complex and subsequently inhibiting bacterial protein synthesis (1–3). The unique site and mode of action make it challenging to develop cross-resistance with other antimicrobial agents. In comparison to vancomycin, linezolid does not necessitate routine monitoring of blood concentrations. Its distribution in tissue and body fluid is favorable, while its high bioavailability allows for convenient administration via both oral and intravenous routes. In recent years, the utilization of linezolid has become more extensive. Common adverse reactions associated with linezolid encompass diarrhea, nausea, headache and thrombocytopenia (4, 5). Although teeth discoloration has been mentioned in the literature manuals as a potential side effect, it is rarely observed in clinical practice. A case involving children was analyzed and discussed, where linezolid-induced teeth discoloration occurred with the shortest possible incubation period. This analysis aims to enhance the expertise of pediatricians and clinical pharmacists in providing pharmaceutical care.

## 2 Case report

A 5-year-old boy was hospitalized with bacterial meningitis and tonsillitis because of fever for 2 days, headache for 1 day, accompanied by vomiting”. The patient presented with an unexplained fever two days ago, with a maximum temperature of 39 ℃, no chills, convulsions, or rashes. The patient presented with symptoms of headache, dizziness, paroxysmal symptoms, accompanied by vomiting 3 times, non-ejaculating, and the vomitus were gastric contents, without any coffee colored appearance. The patient, who had received outpatient cefminox treatment once, was subsequently admitted to the pediatric department due to persistent fever and headache. Blood routine: white blood cell (WBC) 11.17 × 10^9^/L, neutrophilic granulocyte percent (*N*%) 88.4%, C-reactive protein (CRP) 9.38 mg/L, erythrocyte sedimentation rate (ESR) is 35 mm/h. Cerebrospinal fluid biochemistry shows a glucose level of 3.00 mmol/L, chloride level of 123.0 mmol/L, and protein quantification of 0.19 g/L; Cerebrospinal fluid routine reveals clear and transparent appearance with a nucleated cell count of 261.0 × 10^6^/L, consisting of monocytes accounting for 95.8% and multinucleated cells accounting for the remaining 4.2%. The culture of cerebrospinal fluid yielded negative results. He received a prescription for intravenous ceftriaxone at a dose of 1 g (50 mg/kg) every 12 h and linezolid at a dose of 220 mg (10 mg/kg) every eight hours, which lasted for a total duration of fourteen days as part of the antimicrobial treatment plan. Additionally, dexamethasone was prescribed at a dosage of 5 mg every 12 h for a period of four days to suppress inflammatory reactions. On the 4th day of admission, the child did not exhibit any further fever.

**Figure 1:**
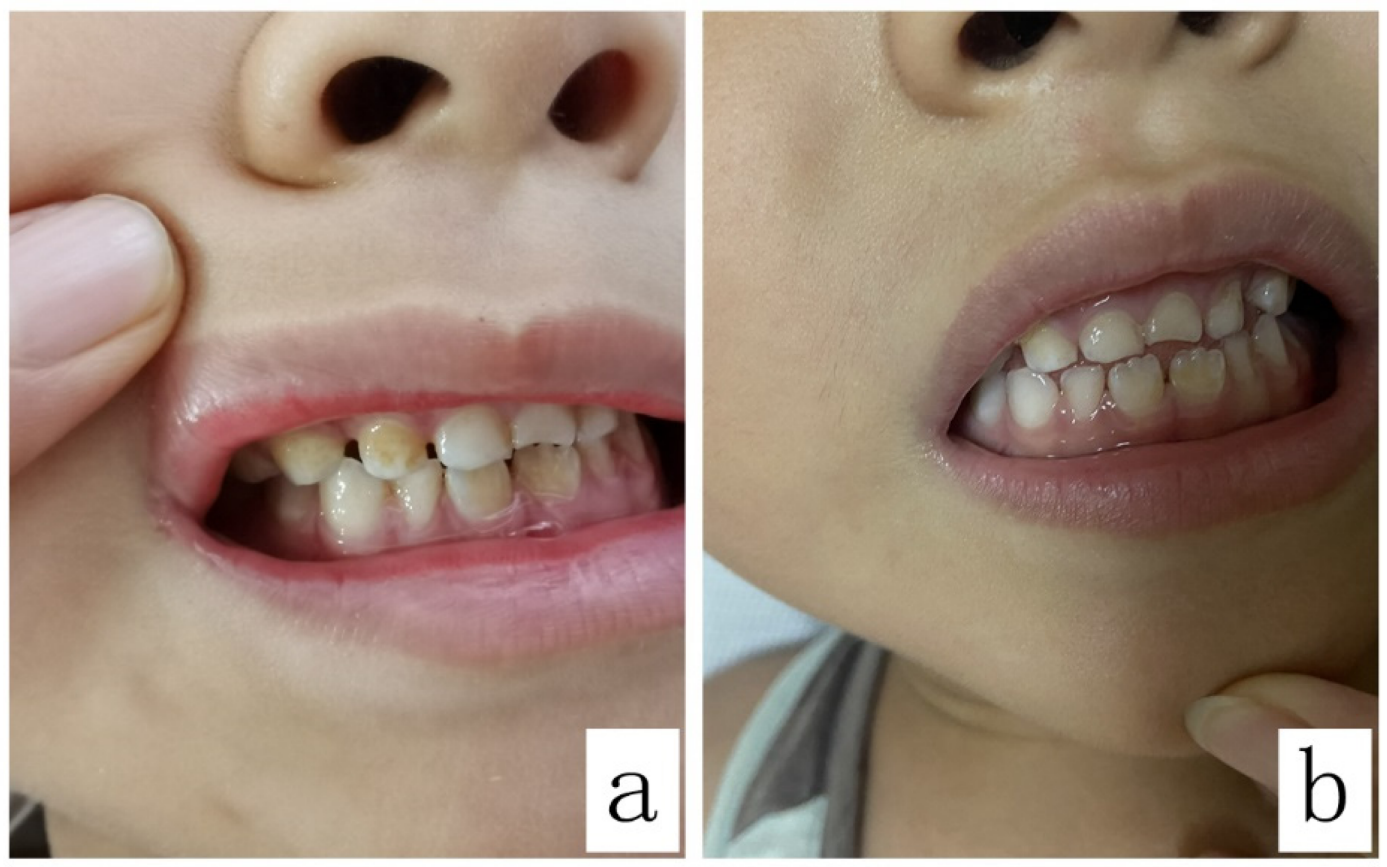
Brown discoloration of the teeth before and after the discontinuation of linezolid: **(a)** shows the teeth discoloration in the patient; **(b)** shows the teeth in the patient 28 days after the discontinuation of linezolid.

The teeth exhibited noticeable brown discoloration (Figure 1) and proved resistant to removal through regular dental hygiene practices on the 5th day of linezolid administration. The clinician consulted the clinical pharmacist on the same day to identify the causative agent of the adverse reaction and determine if a switch to alternative medications was necessary. Since linezolid therapy was well tolerated except for reversible teeth discoloration, the clinical pharmacist recommended continuing its use as the discoloration is expected to be temporary and leave no lasting effects. The clinician accepted the suggestions. After the discontinuance of linezolid for 28 days, the teeth discoloration gradually returned to normal without requiring manual descaling (according to his mother's description, refer to Figure 1 for a corresponding photo).

**Figure 2:**
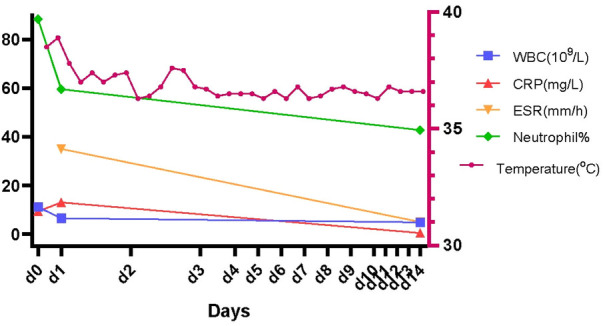
The inflammation findings and temperature of the patient.

After 3 days of ceftriaxone and linezolid application, the patient's temperature remained stable, and there was gradual improvement in inflammation findings. The patient's inflammation findings and temperature after the therapy are illustrated in Figure 2.

During the hospitalization, patient refrained from consuming food and beverages that have the potential to cause teeth discoloration, such as coffee and tea. Linezolid was suspected to be the causative drug due to the chronologic occurrence of the reaction and a lack of association with the patient's concomitant drugs. This adverse reaction was classified as probable correlation with a score of 6 according to the Naranjo Adverse Drug Reaction (ADR) Probability scale shown in Table 1.

**Table 1:** The naranjo adverse drug reaction (ADR) probability scale.

	Yes	No	Do not know	Score
1. Are there previous conclusive reports on this reaction?	1	0	0	1
2. Did the adverse event occur after the suspected drug was administered?	2	−1	0	2
3. Did the adverse reaction improve when the drug was discontinued or a specific antagonist was administered?	1	0	0	1
4. Did the adverse reaction reappear when the drug was readministered?	2	−1	0	0
5. Are there alternative causes (other than the drug) that could have on their own caused the reaction?	−1	2	0	2
6. Did the reaction reappear when a placebo was given?	−1	1	0	0
7. Was the blood detected in the blood (or other fluids) in concentrations known to be toxic?	1	0	0	0
8. Was the reaction more severe when the dose was increased or less severe when the dose was decreased?	1	0	0	0
9. Did the patient have a similar reaction to the same or similar drugs in any previous exposure?	1	0	0	0
10. Was the adverse event confirmed by any objective evidence?	1	0	0	0
Total				6

## 3 Discussion

Considering the significant mortality rate and incidence of sequelae associated with bacterial meningitis, it is crucial to promptly initiate an antibiotic therapeutic regimen in children suspected of having meningitis following lumbar puncture. The initial doses of empiric antibiotic therapy should be administered as follows: Vancomycin (15 mg/kg IV), PLUS Ceftriaxone (50 mg/kg IV) or cefotaxime (100 mg/kg IV) (6, 7). Due to the inability to monitor blood concentration and potential renal damage caused by vancomycin, linezolid is relatively safer and more effective for treating central nervous system infection. In addition, the oral dosage formulation of linezolid, which offers greater convenience for sequential treatment, is typically preferred as an initial therapy for gram-positive bacterial infections in pediatric patients. Therefore, linezolid (10 mg/kg IV q8 h) in combination with Ceftriaxone (50 mg/kg IV q12 h) is recommended as the initial therapy for this child.

Consider administering dexamethasone therapy (0.15 mg/kg IV) to patients with specific risk factors, such as young children (age ≥6 weeks to ≤5 years) or if there is known or suspected *Haemophilus influenzae* infection (e.g., based on Gram stain results) (8). Dexamethasone was administered just with the first dose of empirical antibiotic therapy for 4 days.

The discoloration of teeth can be categorized into two types: extrinsic stains, which are located on the outer surface of the teeth and can be removed through manual descaling; and intrinsic stains, which are attached to the structure of the teeth. The tooth discoloration caused by linezolid is superficial in nature and reversible (9). Tetracyclines cause the other category of discoloration. Apart from reversible teeth discoloration, linezolid therapy is generally well-tolerated without the need for any intervention. The parents of the child should not be overly anxious or seek oral treatment.

The occurrence of teeth discoloration induced by linezolid is rare adverse reaction, but it has been reported in adult and children before (3, 9–16). The literature in Table 2 presents the dental discoloration caused by linezolid. Generally speaking, according to the literature, the incubation period of this adverse reaction is 7 days–8 weeks, regardless of the route of administration. The discoloration can generally fade to normal color within 1 month to 5 months after discontinuation of linezolid. The clinical manifestations of this ADR were similar to those reported in literature, but the occurrence time of ADR in this case was earlier than those reported. To our knowledge, this represents the case report with the shortest incubation period documented worldwide.

**Table 2:** A summary of the literature of teeth discoloration induced by linezolid.

Author	Ages (years)	Gender	Diagnosis	Delivery way	Dosage	Incubation period	Color of teeth discoloration	Time of discoloration fade to normal color
Zou (3)	10	Boy	Osteomyelitis	Intravenously	10 mg/kg q8h	12th day	Yellow	3 months
					10 mg/kg q12h	14th day	Yellow	Not clarified
Agrawal (9)	30	Male	Ulcer on the dorsal aspect of the right forearm	Oral	600 mg twice daily	6th week	Brown	Not clarified
(10)	Mid-20 s	Woman	Active tuberculosis	Not clarified	600 mg daily	8th week	Brown discoloration near the gumline	Not clarified
Santos (11)	9	Girl	Surgical-site infection	Oral	30 mg/kg/d every 8 h	4th week	Linear brownish	4 weeks
Petropoulou (12)	5	Boy	Severe pneumonia	Intravenously	30 mg/kg/day q8h	3rd week	Brown discoloration	2 months
	8	Boy	Severe skin infection of the left foot	Intravenously	30 mg/kg/day q8h	7th day	Brown discoloration	1 months
	14	Girl	Left orbital cellulitis	Intravenously	30 mg/kg/day q8h	3rd week	Brown discoloration	2 months
Ma (13)	8	Girl	Bacteremia and polyarthritis	Oral	30 mg/kg/d in 3 divided doses	1st week	Linear brownish discoloration	4 weeks
Matson (14)	11	Girl	Cellulitis of the toe localized to the first distal phalange	Oral	600 mg twice/day	3rd week	Brown discoloration	Not clarified
Xu (15)	Not clarified	Not clarified	Infective endocarditis	Oral	<11Y, 10 mg/kg q8 h;≥11Y, 10 mg/kg q12h	5th month	Brown discoloration	5 months
Chavanda (16)	35	Male	MSSA and Mycobacterium abscesses infection	Not clarified	600 mg OD	16th day	Dark discoloration	3 weeks
This work	5	Boy	bacterial meningitis	intravenously	10 mg/kg q8h	5th day	Brown discoloration	28 days

Unfortunately, the specific mechanism underlying teeth discoloration caused by linezolid remains unclear. Diverse perspectives exist regarding the mechanism of this discoloration. Some propose that linezolid can alter the oral microbiota, leading to an overgrowth of certain species in the genus *Neisseria*, *Porphyromonas* and *Streptococcus* dominant. The metabolic activity of these special microbiota can generate hydrogen sulfide, which combines with iron in saliva to form insoluble iron salts rich in calcium and phosphate contents. These deposits then accumulate on the teeth surface, forming distinctive dental plaques (17). Others argue that linezolid exhibits altered affinity towards oral structures.

From Table 2, it can be seen that there were 8 cases of teeth discoloration caused by linezolid in children, while only 3 cases were reported in adults. The incidence in children is significantly higher than that in adults. The reason for this difference may be that the microorganisms in children's oral cavity are more susceptible to the influence of linezolid. Perhaps that children's teeth are more susceptible to development disturbances during mineralization phase of teeth formation, and permanent dentition is more susceptible to disturbances in mineralization by drugs (18). The child in this case is 5 years old and in the stage of teeth formation, and the above theory applies.

In this case, on the 5th day of treatment with the normal dosage of linezolid, teeth discoloration occurred. Due to the patient's good tolerance and no other discomfort except for teeth discoloration, the anti-infection treatment was completed. During this period, the discoloration of the child's teeth persisted until 28 days after discontinuing linezolid, when the teeth faded to normal, which consistent with literature, except for a short incubation period. Unfortunately, due to a lack of experience, the child in this case did not receive active intervention after the ADR occurred. In future clinical practice, similar situations may arise, and probiotics may be taken orally for a certain period of time to observe the progression of ADR and explore potential mechanisms.

## 4 Conclusion

With the increasing utilization of linezolid, especially in pediatric patients with Gram-positive bacterial infections, rare ADRs associated with linezolid are being increasingly identified. This case serves as a reminder to pediatricians and clinical pharmacists regarding the importance of exercising caution when prescribing this medication. It is crucial to promptly provide medication education to parents or caregivers about the potential reversibility and estimated recovery time of these ADRs in order to alleviate their anxiety.

## Data Availability

The original contributions presented in the study are included in the article/Supplementary Material, further inquiries can be directed to the corresponding authors.
